# 2957. Decreased microbial diversity and baseline intestinal inflammation are associated with worse outcome in aged, female mice infected with *Clostridioides difficile*

**DOI:** 10.1093/ofid/ofad500.196

**Published:** 2023-11-27

**Authors:** Nhu Nguyen, Kimberly Vendrov, Lisa Abernathy-Close, Michael Dieterle, Vincent B Young

**Affiliations:** University of Michigan, Ann Arbor, MI; University of Michigan, Ann Arbor, MI; University of Michigan, Ann Arbor, MI; University of Michigan, Ann Arbor, MI; University of Michigan Medical School, Ann Arbor, MI

## Abstract

**Background:**

*C. difficile* infection is more common and severe in people over 65-years old. Understanding the pathogenesis within the triangle of host, pathogen, and the microbiota in aged population will help decrease the disease severity.

**Methods:**

Young (3-month-old) and aged (22 to 25-month-old) male and female C57BL/6 mice were treated with 0.5g/L cefoperazone for ten days to render them susceptible to *C. difficile* infection. Antibiotic-treated mice were challenged with 10^3^ to 10^4^ spores of *C. difficile* via oral gavage. Three *C. difficile* strains were selected for challenge. These strains vary in the severity of disease observed in young, cefoperazone-treated mice; CD630 (mild disease), R20291 (moderate), and VPI10463 (severe). Mice were monitored for weight loss and clinical signs of disease following challenge. The degree of intestinal inflammation was determined by fecal lipocalin-2 measurement. The microbiota of animals before challenge was monitored by 16S rRNA-encoding gene sequence analysis.

Mouse model of C. difficile infection

**Figure:**
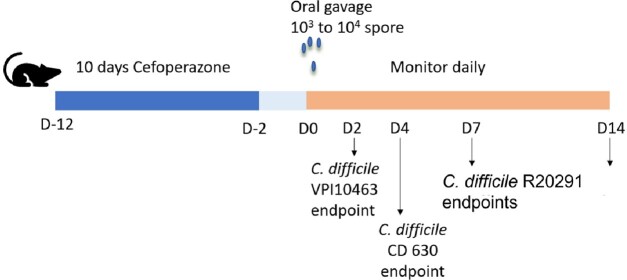

**Results:**

Overall, there was no significant difference in weight loss between young and aged mice following challenge. However, aged female mice lost significantly more weight than male mice when infected with *C. difficile* CD630 (p = 0.031) and R20291 (p = 0.0027). Microbiota analysis revealed that alpha diversity was lower in female aged mice compared to male aged mice. No sex differences were noted in young mice. Measurement of fecal lipocalin revealed that aged female mice had baseline inflammation not seen in aged male mice (p = 0.02). Finally, we found a strong negative correlation between species richness (Sobs) and maximum weight loss (R-squared=0.74) in aged mice. Lipocalin-2 levels were moderately correlated with maximum weight loss (R-squared=0.51).

**Figure 1.**
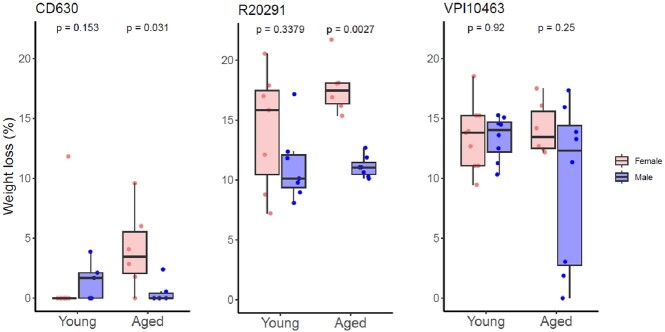
Weight loss comparing between male and female mice across age group and C. difficile strains.

**Figure 2.**
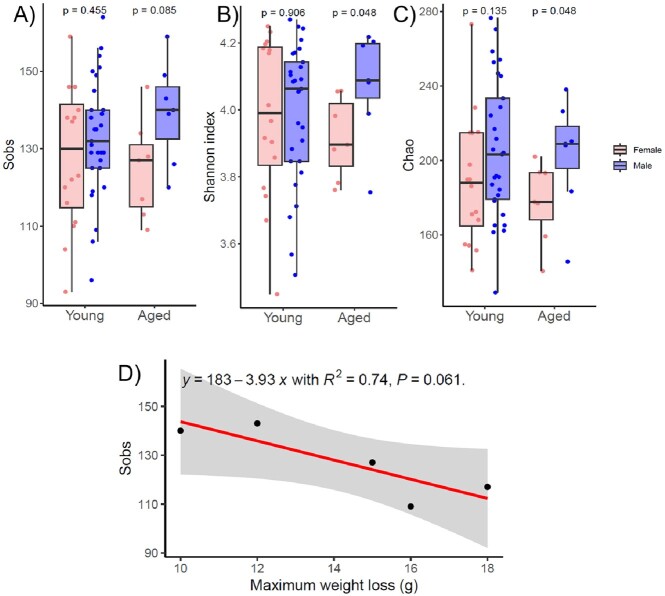
The microbiota of young and aged mice before challenging with cefoperazone. Alpha diversity indexes A) Sobs, B) Shannon index, C) Chao when comparing between male and female mice across age group. D) Linear regression between species richness (Sobs) and maximum weight loss from aged mice group.

**Conclusion:**

The combination of a predisposed low diversity microbiota and baseline inflammation gut may contribute to a more severe outcome for female aged mice infected with *C. difficile*.

**Figure 3.**
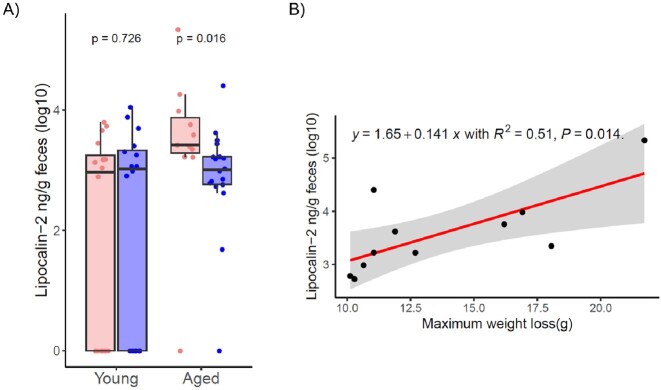
The degree of baseline intestinal inflammation in young and aged mice. A) Lipocalin-2 measurement at baseline comparing between male and female mice, B) linear regression between fecal lipocalin-2 at baseline and maximum weight loss in aged mice group.

**Disclosures:**

**Vincent B. Young, MD/PhD**, ASM: Senior Editor|Debiopharm: Advisor/Consultant|Vedanta Biosciences: Advisor/Consultant

